# Advances in mitochondrial dysfunction in radiation tissue injury

**DOI:** 10.3389/fphys.2025.1660330

**Published:** 2025-08-20

**Authors:** Jianhuang Rong, Qiujie Yu, Guilin Huang, Yueyue Wang, Nini Zhang

**Affiliations:** ^1^ Department of Oral Maxillofacial Surgery, School and Hospital of Stomatology, Zunyi Medical University, Zunyi, China; ^2^ Department of Laboratory Medicine, The Third Affiliated Hospital of Zunyi Medical University (The First People’s Hospital of Zunyi), Zunyi, China; ^3^ Zunyi Medical University, Zunyi, China

**Keywords:** mitochondrial dysfunction, radiation injury, oxidative stress, mitophagy, apoptosis, energy metabolism

## Abstract

Radiation-induced tissue injury is a major limitation in cancer radiotherapy, often leading to collateral damage in healthy tissues. While the nucleus has long been considered the principal target of ionizing radiation, emerging evidence underscores the pivotal role of mitochondria in mediating radiation-induced damage. This review provides a comprehensive overview of mitochondrial dysfunction in various irradiated tissues, including the intestine, hematopoietic system, heart, lung, brain, and skin. Key mitochondrial alterations—such as disrupted dynamics, impaired energy metabolism, excessive reactive oxygen species (ROS) production, and activation of apoptotic and senescence pathways—are highlighted as central mechanisms underlying radiation pathology. Additionally, we summarize the involvement of crucial signaling pathways such as AMP-activated protein kinase/peroxisome proliferator-activated receptor gamma coactivator 1-alpha (AMPK/PGC-1α),nuclear factor erythroid 2–related factor 2/antioxidant response element/mitochondrial transcription factor A (Nrf2/ARE/TFAM), and NOD-like receptor family pyrin domain containing 3 (NLRP3) inflammasome in regulating mitochondrial responses to radiation stress. A deeper understanding of mitochondrial involvement provides novel avenues for radioprotection and therapeutic interventions in oncology.

## 1 Introduction

Radiotherapy is one of the primary modalities for cancer treatment alongside surgery, and despite significant advancements in precision radiotherapy, it is still difficult to avoid radiation collateral damage to normal peri-cancer tissues during radiotherapy (Bourdais et al., 2021). Up to now, only two drugs, amifostine and palifermin, have been approved by the U.S. Food and Drug Administration (FDA) as radioprotective agents (Zivkovic Radojevic et al., 2023), but their severe toxic side effects have significantly limited their clinical application. In addition to the off-target effects already confirmed by radiotherapy, radiation injury is also a concern in other situations, such as in radiation public health incidents and among astronauts on long-term space flights. In these environments, the risks resulting from radiation exposure are similar to those in radiotherapy (DiCarlo, 2021; Rudolf and Hood, 2024).

**FIGURE 1:**
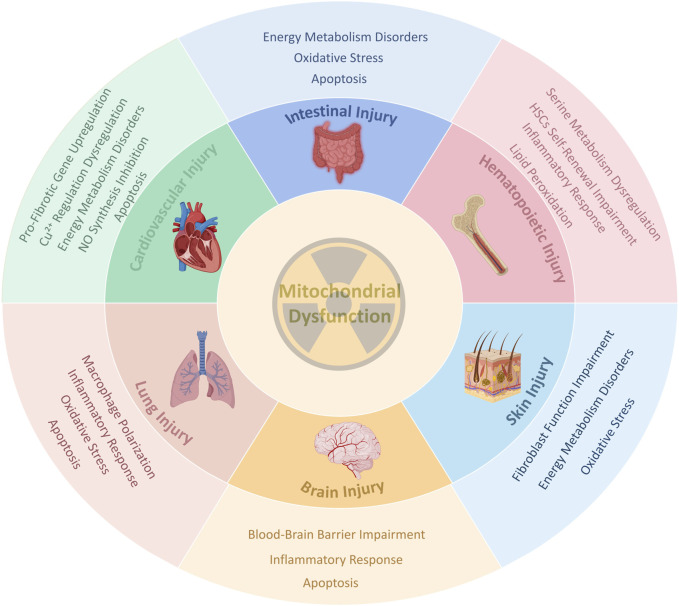
A schematic diagram of the core role of Mitochondrial Dysfunction in radiation-induced tissue injury. This figure shows a series of common responses such as oxidative stress, inflammation, apoptosis, energy crisis, and tissue-specific pathological processes triggered by radiation-induced mitochondrial dysfunction in different organs (intestine, hematopoietic system, cardiovascular system, lungs, brain, and skin), ultimately leading to tissue damage. Created in BioRender. rong, j. (2025) https://BioRender.com/ca6330z.

Therefore, it is imperative to investigate the underlying mechanisms of radiation-induced tissue injury and to develop novel therapeutic strategies. While the nucleus has long been considered the primary target of radiation damage, growing evidence indicates that mitochondria are also critical subcellular organelles affected by radiation (Szumiel, 2015; Calarco et al., 2021). Under stress conditions such as radiation exposure, mitochondria are highly susceptible to damage and may act as intracellular danger signals (Li et al., 2023). Indeed, many common human diseases—including aging, ischemia-reperfusion injury, and neurodegeneration—exhibit characteristics similar to those observed in radiation-induced mitochondrial dysfunction, such as mitochondrial DNA (mtDNA) damage, oxidative stress, and impaired energy metabolism. Fundamental research has confirmed a strong association between mitochondrial dysfunction and these pathologies (Wen et al., 2023). Based on these findings, this review aims to summarize the recent evidence regarding radiation-induced mitochondrial dysfunction, including related studies and emerging insights into mitochondrial damage across various irradiated tissues (Figure 1). The goal is to further elucidate the connection between mitochondrial dysfunction and radiation injury, and to provide novel perspectives for the prevention and treatment of radiation-induced tissue injury.

## 2 Functions of mitochondria in cells

The mitochondrion is a double-membrane-bound organelle commonly referred to as the cell’s “powerhouse”, with energy production as its primary function. It is responsible for cellular respiration and the majority of adenosine triphosphate (ATP) generation in eukaryotic cells (Rossmann et al., 2021). Mitochondrial oxidative phosphorylation (OXPHOS) and the electron transport chain (ETC) operate in close coordination to produce energy. The ETC utilizes reducing equivalents derived from nutrients to pump protons across the inner mitochondrial membrane, creating a proton gradient. This gradient drives ATP synthase to convert adenosine diphosphate (ADP) into ATP through OXPHOS, thereby enabling the conversion and storage of biochemical energy to support a wide range of cellular processes (Irene and Leonid A, 2022).

In addition to energy production, mitochondria play essential roles in the regulation of programmed cell death, intracellular signaling pathways, calcium homeostasis, redox balance, and the biosynthesis of key macromolecules such as amino acids, nucleotides, and fatty acids (Onishi et al., 2021). Therefore, mitochondrial dysfunction is not limited to reduced intracellular ATP levels; it also includes the accumulation of ROS and reactive nitrogen species (RNS), calcium overload, and other mitochondrial disturbances. Unlike other organelles, mitochondria possess their own genetic material—mtDNA—which encodes several essential subunits of the respiratory chain complexes and functions independently of nuclear DNA. Under normal physiological conditions, mtDNA is packaged with specific proteins into nucleoid structures that are stably maintained within the mitochondria. However, various physiological and pathological stressors can induce mtDNA damage. The accumulation of damaged mtDNA impairs mitochondrial function, thereby disrupting cellular homeostasis and contributing to disease pathogenesis (Liao et al., 2022; Harrington et al., 2023).

## 3 Radiation-induced manifestations of mitochondrial dysfunction

### 3.1 Alterations in mitochondrial dynamics

**FIGURE 2:**
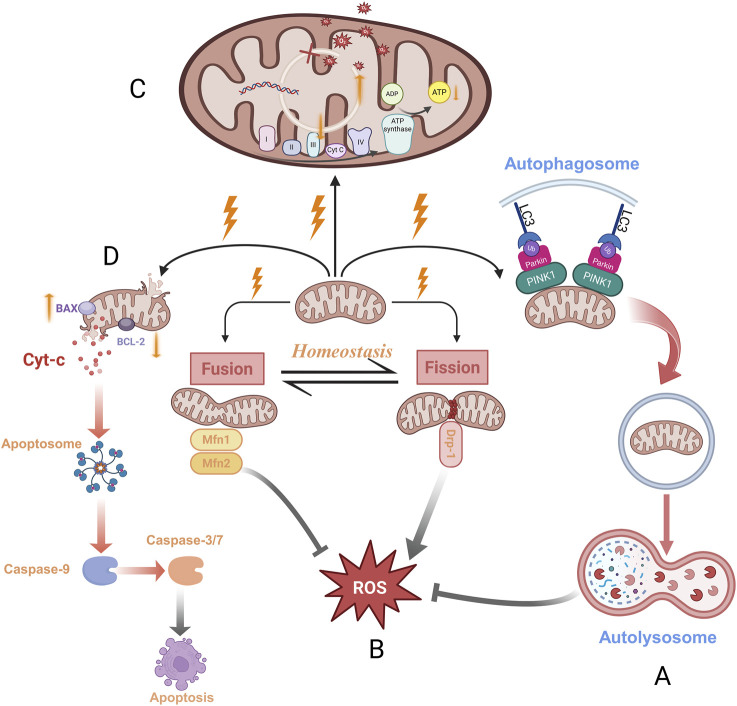
Schematic diagram of radiation - induced mitochondrial dysfunction manifestations. **(A,B)** The relationship between mitochondrial dynamic balance (autophagy, fission, fusion) and ROS generation under radiation stress; **(C)** Presents the vicious cycle process of abnormal mitochondrial energy metabolism: ROS elevation → mtDNA damage → reduced ETC activity → further ROS surge. **(D)** Presents the radiation - induced mitochondrial - pathway - dependent apoptosis process. Cyt-c binds with intracellular adaptor proteins to form the apoptosome, which activates procaspase-9, followed by the activation of caspase-3 and caspase-7, ultimately initiating a caspase cascade that drives mitochondrial pathway-dependent apoptosis. Bcl-2 family of proteins controls mitochondrial apoptosis and initiates cell death through the pro-apoptotic activity of BAX and Bcl-2 homologous BAK on the mitochondrial outer membrane. The antiapoptotic BCL-2 protein directly binds to BAX and BAK to prevent their oligomerization, ensuring cell survival. Created in BioRender. rong, j. (2025) https://BioRender.com/jb2dzbi.

Mitochondrial dynamics refers to the highly regulated processes of mitochondrial autophagy (mitophagy), fission and fusion (Figures 2A,B). Ionizing radiation induces mitochondrial swelling, depolarization of the mitochondrial membrane potential (MMP), elevated ROS levels, and mtDNA damage; these stressors collectively activate mitophagy as a compensatory mechanism to preserve mitochondrial homeostasis (Wang and Liu, 2024). Radiation-induced mitochondrial dysfunction often increases membrane permeability, releasing mtDNA into the cytoplasm, which activates the cyclic GMP–AMP synthase–stimulator of interferon genes (cGAS–STING) pathway and triggers an inflammatory response (Guilbaud et al., 2024). PTEN-induced kinase 1 (PINK1)/Parkin-mediated mitophagy can attenuate this inflammatory cascade by eliminating damaged mitochondria, thereby reducing mtDNA mutation accumulation and suppressing STING pathway activation (Sliter et al., 2018). In response to cellular stress such as radiation, a decline in MMP leads to PINK1 accumulation on the outer membrane, which recruits and activates Parkin. Activated Parkin ubiquitinates outer mitochondrial membrane proteins, promoting autophagosome formation through recruitment of microtubule-associated protein 1 light chain 3 (LC3). LC3, via its LC3-interacting region (LIR), facilitates mitochondrial sequestration into autolysosome for degradation (Lazarou et al., 2015; Yamada et al., 2019).

Beyond autophagy, mitochondria continuously undergo morphological changes—including fission and fusion—in response to shifts in metabolic demand or cellular stress (Khacho et al., 2016). These dynamic processes are vital for maintaining mitochondrial health, and disruption of this balance can lead to mitochondrial dysfunction. Dynamin-related protein 1 (DRP1) is essential for mitochondrial fission, while mitofusin 1 and 2 (MFN1 and MFN2) mediate mitochondrial fusion (Basha et al., 2022). Radiation disrupts this fission–fusion balance, and excessive mitochondrial fission increases ROS production, which further disrupts mitochondrial dynamics—forming a vicious DRP1–mitochondrial ROS (mtROS) cycle (Yamada et al., 2019). Given that mitochondria are major sources of intracellular ROS (Jonathan et al., 2022), understanding the contribution of mitochondrial dynamics to radiation-induced oxidative stress may offer new therapeutic targets. Regulation of mitochondrial dynamics not only modulates inflammation but also mitigates oxidative stress, thereby reducing the severity of radiation-induced tissue damage.

### 3.2 Abnormal mitochondrial energy metabolism

Mitochondria are essential for eukaryotic life, providing cellular energy through the tricarboxylic acid (TCA) cycle, ETC, and OXPHOS, and are critical for maintaining normal physiological processes (Acin-Perez et al., 2023). Abnormal mitochondrial metabolism has been strongly implicated in a wide range of diseases, including type 2 diabetes, cancer, aging, and organ fibrosis (Xu et al., 2021; Pavlova et al., 2022; Rabbani et al., 2022). Radiation acutely elevates mtROS levels, aggravates mtDNA damage, and reduces the transcription of mtDNA-encoded ETC subunits, ultimately resulting in decreased ETC activity. In turn, impaired ETC function promotes further mtROS production, establishing a self-perpetuating cycle of oxidative stress (Ait-Aissa et al., 2023) (Figure 2C). Persistent oxidative stress leads to an imbalance in the intracellular antioxidant defense system, further exacerbating mitochondrial injury and enhancing ROS generation—a vicious cycle that contributes to cellular dysfunction (Rogov et al., 2021; Wang D.-K. et al., 2022). Mitochondria are responsible for generating approximately 95% of the energy required for cellular function, making them indispensable for maintaining both metabolic homeostasis and supporting cellular repair processes (Onishi and Okamoto, 2021). To some extent, radiation-induced DNA damage repair is an energy-consuming process that requires an adequate supply of ATP for DNA repair and cell survival under stress conditions (Qin et al., 2015). Thus, radiation-induced disruption of mitochondrial energy metabolism not only intensifies oxidative stress in irradiated tissues, but also impairs the regenerative capacity of damaged cells.

### 3.3 Mitochondria-mediated cell death and senescence

Radiation can directly damage cellular DNA, causing base modifications and DNA strand breaks, which in turn trigger cell death. It can also indirectly impair mitochondrial function by increasing ROS levels, thereby activating the mitochondria-dependent apoptotic pathway (Averbeck and Rodriguez-Lafrasse, 2021). Damaged mitochondria promote the release of cytochrome c (Cyt-c), which amplifies the apoptotic response (Liu et al., 2021). A decline in MMP is a hallmark of early apoptosis and facilitates the release of Cyt-c into the cytoplasm. Cyt-c triggers apoptosome formation and activates caspase cascades that drive intrinsic apoptosis (Kong et al., 2023; Yan et al., 2023). The B-cell lymphoma 2 (Bcl-2) family tightly regulates this process; pro-apoptotic Bcl-2-associated X protein (BAX) and Bcl-2 homologous antagonist/killer (BAK) promote mitochondrial outer membrane permeabilization, whereas anti-apoptotic Bcl-2 inhibits this cascade (Wolf et al., 2022) (Figure 2D). In addition to the classical apoptotic pathway, Mitochondrial dysfunction activates necroptotic signaling molecules, potentially through the activation of calcium/calmodulin-dependent protein kinase II (CaMK II), which regulates the opening of the mitochondrial permeability transition pore (mPTP) and drives the progression of necroptosis (Yi et al., 2023). Moreover, radiation can induce intracellular iron overload. Free iron activates mitoferrin-2 (Mfrn2) on the mitochondrial membrane, and Mfrn2-mediated transport of cytosolic free iron into the mitochondria leads to mitochondrial iron overload and excessive ROS production, ultimately resulting in lipid peroxidation and ferroptosis (Wang L. et al., 2022; Zhou et al., 2022). Radiation-induced mitochondrial dysfunction also activates the senescence pathway, particularly through the p53–Cyclin-dependent kinase inhibitor 1 (p21) signaling axis. Kim et al. demonstrated that mitochondrial dysfunction elevates p21 expression, suppresses cell proliferation, and disrupts the cell cycle via p53-dependent regulation (Ibragimova et al., 2024). Upon activation, the p53 regulates several downstream genes, including p21 and p16, both of which are cyclin-dependent kinase inhibitors. These molecules induce cell cycle arrest and promote cellular senescence by inhibiting cyclin-dependent kinases (Kumari and Jat, 2021).

Collectively, radiation-induced mitochondrial dysfunction leads to excessive cell death and premature senescence, disrupting cellular homeostasis and ultimately contributing to functional decline in irradiated tissues.

## 4 Key signaling pathways involved in the regulation of mitochondrial activity

**FIGURE 3:**
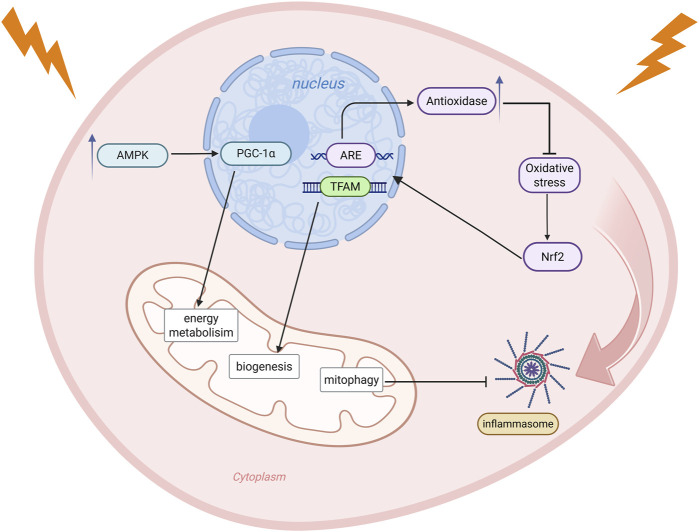
Radiation stress activates multiple mitochondria-related signaling pathways that coordinate cellular responses to damage. Under energy depletion, the AMPK/PGC-1α pathway is activated, enhancing PGC-1α expression and improving cellular energy metabolism. Oxidative stress stimulates the Nrf2 pathway, which translocates to the nucleus, activates ARE, induces antioxidase expression, and promotes TFAM-mediated mitochondrial biogenesis to alleviate oxidative stress. Mitophagy inhibits activation of the NLRP3 inflammasome signaling pathway, thereby suppressing inflammation. Created in BioRender. rong, j. (2025) https://BioRender.com/a8plvxf.

In radiation-induced tissue injury, mitochondria are not only a primary target of the initial damage but also serve as a central platform for signal regulation. Multiple signaling pathways are involved in the cellular response to and repair of radiation stress by regulating mitochondrial energy metabolism, oxidative stress responses, inflammatory responses, and other processes (Figure 3).

### 4.1 The AMPK/PGC-1α signaling pathway

AMPK is a key regulator of cellular energy sensing, and its activation upregulates the expression of PGC-1α, a key transcription factor for mitochondrial biogenesis. This, in turn, promotes the restoration of mitochondrial number and function, thereby improving cellular energy metabolism (Herzig and Shaw, 2018; Liu et al., 2024). Under radiation stress, this pathway helps alleviate energy depletion and maintain cell survival and mitochondrial homeostasis, acting as one of the critical protective mechanisms.

### 4.2 The Nrf2-ARE/TFAM signaling pathway

Nrf2-mediated signaling is a crucial defense mechanism against oxidative stress (Shen et al., 2023), its activity is significantly elevated under radiation-induced oxidative stress (Rai et al., 2024). Multiple studies have demonstrated the involvement of Nrf2 in the regulation of MMP, ATP production, respiration rate, and OXPHOS efficiency (Dinkova-Kostova and Abramov, 2015). Upon activation, Nrf2 initiates an Antioxidant Response Element (ARE) -mediated transcriptional program to induce the expression of several antioxidant enzymes and simultaneously enhances the expression of the Mitochondrial transcription factor A (TFAM), thereby promoting mitochondrial biogenesis (Ryoo and Kwak, 2018). This pathway helps mitigate radiation-induced oxidative damage and facilitates mitochondrial repair.

### 4.3 The NLRP3 inflammasome signaling pathway

When mitochondria are exposed to radiation, they enter a highly oxidized state and produce large amounts of ROS, which act as signals to activate the NLRP3 inflammasome and trigger an inflammatory response (Ortiz et al., 2015). Recent studies have shown that mitophagy can serve as an intervention target for NLRP3 inflammasome activation, and the promotion of mitophagy inhibits the activation of NLRP3 inflammasome (Ding et al., 2024). In addition to these signaling pathways, radiation has also been shown to trigger inflammatory responses by impairing mitochondrial function, subsequently activating the nuclear factor kappa B (NF-κB) signaling pathway (Ait-Aissa et al., 2024). A deeper understanding of the interactions among these signaling pathways in the future will help reveal the central regulatory role of mitochondria in radiation injury and provide a theoretical basis for the development of targeted therapeutic strategies.

## 5 Mitochondrial dysfunction in radiation tissue injury

### 5.1 Mitochondrial dysfunction in intestinal injury

The intestinal epithelium is among the most radiosensitive tissues and is frequently subjected to incidental radiation exposure during the therapeutic management of abdominal and pelvic malignancies. Such exposure often results in a broad spectrum of injuries, including villous atrophy, inflammation, ulceration, hemorrhage, and pain. Clinically, acute gastrointestinal symptoms such as nausea, vomiting, and diarrhea may manifest early during radiotherapy, while chronic complications such as mucosal atrophy, fibrosis, and impaired absorptive function frequently develop during the later stages (Loge et al., 2020; Lu et al., 2023). Accumulating evidence indicates that mitochondrial dysfunction plays a pivotal role in the pathogenesis of radiation-induced intestinal injury. Zhao et al. demonstrated that radiation significantly suppressed the activity of mitochondrial respiratory chain complexes in murine intestinal tissue, concomitantly decreasing intracellular ATP levels. These alterations were accompanied by downregulation of mitochondrial gene expression and copy number, increased oxidative stress and inflammatory responses, and subsequent disruption of intestinal barrier integrity (Zhao et al., 2020).

Rapamycin (RAPA), a pharmacological activator of autophagy, was shown to ameliorate mitochondrial respiratory function and attenuate the accumulation of ROS, reactive nitrogen species (RNS), malondialdehyde (MDA), and hydrogen peroxide (H_2_O_2_), thereby mitigating oxidative stress (Qu et al., 2020). Similarly, He et al. reported radiation-induced ultrastructural abnormalities in rat intestinal mitochondria, including swelling, cristae disruption, and vacuolization, as well as reductions in the activities of ETC complexes I–V and in ATP production. These observations indicated significant mitochondrial impairment. Moreover, radiation decreased the expression of mitophagy-related proteins Parkin and PINK1, suggesting suppression of mitochondrial quality control mechanisms (He et al., 2025). In a separate study, mannan oligosaccharide (MOS) was shown to exert a radioprotective effect on intestinal tissues by activating toll-like receptors (TLRs) and enhancing mitochondrial ETC activity. This protective effect was mediated through modulation of the NF-κB, p38, and c-Jun N-terminal kinase (JNK) signaling pathways, upregulation of antioxidant enzymes, and inhibition of cardiolipin peroxidation within the mitochondrial inner membrane (Sanguri and Gupta, 2021). Qiu et al. further demonstrated that radiation-induced mitochondrial dysfunction leads to excessive ROS generation, particularly hydroxyl radicals (•OH), which contribute to oxidative stress and apoptosis. The underlying mechanisms included decreased MMP, Cyt-c release, and activation of caspase-9, caspase-3, and poly (ADP-ribose)polymerase (PARP). Hydrogen treatment exhibited protective effects by maintaining MMP, reducing ROS production, and regulating the expression of apoptosis-related proteins (Qiu et al., 2020).

Collectively, these findings suggest that mitochondrial dysfunction serves as a central mediator in radiation-induced intestinal injury. On the one hand, impaired ATP production compromises epithelial cell repair and functional maintenance; on the other hand, elevated oxidative stress leads to further molecular damage, including DNA, proteins, and lipids. Simultaneously, activation of the mitochondrial apoptotic pathway results in excessive epithelial cell death and loss of mucosal barrier integrity, thus establishing a self-perpetuating cycle that exacerbates intestinal injury.

### 5.2 Mitochondrial dysfunction in hematopoietic system injury

Conventional pelvic radiotherapy is the key treatment for advanced cervical and rectal cancer. However, it inadvertently exposes regions rich in hematopoietic stem cells (HSCs)—such as the iliac bone, sacrum, proximal femur, and lower lumbar spine—to ionizing radiation. HSCs are highly sensitive to radiation, and damage to these areas often results in severe hematological toxicity, which represents a primary cause of radiation-induced bone marrow failure. In recent years, the pivotal role of mitochondria in regulating HSCs fate has garnered increasing attention (Luis et al., 2020; Filippi, 2023). It has been reported that higher mitochondrial mass is positively correlated with the maintenance of HSCs self-renewal capacity (Takihara et al., 2019). Under radiation stress, HSCs exhibit increased frequency and duration of mPTP opening, elevated ROS levels, electron leakage, and reduced MMP, collectively contributing to apoptosis (Bonora et al., 2022). The Nynrin gene is highly expressed under both physiological and radiation-induced stress conditions, and regulates mitochondrial homeostasis by binding to the peptidyl-prolyl cis-trans isomerase F (Ppif) promoter to inhibit the transcription of cyclophilin D (CypD), thereby suppressing mPTP opening, decreasing ROS production, and preserving HSCs dormancy and self-renewal. In contrast, Nynrin deficiency leads to CypD overexpression and mitochondrial dysfunction, rendering HSCs more vulnerable to radiation damage, whereas Nynrin overexpression alleviates these effects (Zhou et al., 2024). Additionally, MMP serves as a marker for distinguishing between quiescent and activated long-term HSCs (LT-HSCs): MMP-high LT-HSCs rely on glycolysis and possess an intact mitochondrial network, whereas MMP-low LT-HSCs exhibit lower mitochondrial activity. These findings indicate that the functional state of mitochondria critically influences HSCs quiescence, activation, and differentiation (Wu et al., 2024).

Radiation also induces the release of mtDNA into the cytoplasm, thereby activating the cGAS signaling pathway and contributing to bone marrow injury. Guan et al. demonstrated that radiation-induced bone marrow damage can be partially mitigated by preventing mtDNA release or blocking cGAS activation (Guan et al., 2023). Moreover, radiation promotes lipid peroxidation and exacerbates mitochondrial oxidative stress. Caffeic acid (CA), a natural antioxidant and specific 5-lipoxygenase (5-LOX) inhibitor, has been shown to reduce oxidative damage and apoptosis in HSCs, thereby exerting a protective effect on hematopoiesis (Wang X. et al., 2021). Increasing attention has also been paid to the role of mitochondrial serine catabolism in radiation-induced myelosuppression. Radiation significantly reduces serum serine levels, whereas supplementation with exogenous serine attenuates ferroptosis and hematopoietic suppression in HSCs, improving survival in murine models. Importantly, this protective effect was abolished in serine hydroxymethyltransferase 2 (SHMT2)-deficient mice, highlighting the critical role of mitochondrial serine metabolism (Du et al., 2024).

It can be seen that regulation of mPTP, maintenance of MMP, inhibition of mtDNA cytoplasmic release and oxidative stress, and improvement of mitochondrial metabolism all play key roles in alleviating radiation-induced hematopoietic system injury. In the future, how to systematically integrate these mitochondrial targets for more effective radiation injury intervention still needs to be explored in depth.

### 5.3 Mitochondrial dysfunction in cardiovascular injury

Radiotherapy not only increases the risk of radiation-induced cardiac injury (Wang K.-X. et al., 2023), but also significantly elevates the incidence of coronary and peripheral atherosclerotic disease (Carlson et al., 2021). However, the molecular mechanisms underlying these changes remain incompletely understood. In recent years, numerous studies have emphasized the pivotal role of mitochondrial dysfunction in the pathogenesis of radiomyocardial fibrosis (RIMF). The structural and functional integrity of mitochondria, which serve as the primary energy source for cardiomyocytes, is essential for maintaining myocardial contraction and relaxation. Radiation disrupts MMP, increases membrane permeability and matrix osmotic pressure, leading to mitochondrial swelling, rupture, and dysfunction. This, in turn, impairs myocardial energy supply, exacerbates tissue injury, and drives the progression of RIMF (Ren et al., 2024). Xu et al. found that after 5 months of exposure to radiation, differentially expressed proteins in the mouse heart were enriched in the “cardiac fibrosis” and “energy metabolism” pathways, with impaired OXPHOS and ATP synthesis identified as key drivers of fibrosis (Xu et al., 2021). Different radiation doses have distinct effects on mitochondrial morphology in vascular endothelial cells: high doses (≥1 Gy) induce mitochondrial fragmentation, whereas low doses (<1 Gy) promote mitochondrial elongation and fusion. These findings suggest that mitochondrial morphological plasticity is closely associated with the cellular response to radiation (Wang L. et al., 2023). Radiation-induced mitochondrial dysfunction promotes fibrosis by activating pro-fibrotic genes, such as transforming growth factor-beta (TGF-β), inhibiting the activity of matrix metalloproteinases (MMPs), and enhancing extracellular matrix (ECM) synthesis and deposition. Concurrently, damaged cardiomyocytes release growth factors such as platelet-derived growth factor (PDGF) and connective tissue growth factor (CTGF), which recruit and activate fibroblasts, inducing their transformation into myofibroblasts and stimulating collagen synthesis, thereby further promoting the fibrosis process (Yu et al., 2023).

Radiation also induces mitochondrial calcium (Ca^2+^) overload in endothelial cells, mediated via the mitochondrial calcium uniporter (MCU), which forms a positive feedback loop with superoxide, causing mtDNA damage and inhibiting nitric oxide (NO) synthesis, thus exacerbating vascular endothelial dysfunction (Ait-Aissa et al., 2022). Pravastatin protects endothelial function and attenuates radiation-induced oxidative stress by inhibiting mitochondrial superoxide production, mtDNA damage, ETC dysfunction, and the expression of inflammatory factors (NF-κB p65, NF-κB p50, TNF-α) (Ait-Aissa et al., 2023). Moreover, a study using human induced pluripotent stem cell-derived engineered heart tissues exposed to γ-rays showed that the mitochondrial-specific antioxidant MitoTempo improved mitochondrial function, reduced ROS, and partially rescued cardiomyocyte contractile dysfunction (Cao X. et al., 2024). The mitochondrial inner membrane protein protein tyrosine phosphatase mitochondrial 1 (PTPMT1) plays a critical role in cardiolipin synthesis and cardiac development. Radiation downregulates PTPMT1 expression in induced pluripotent stem cell-derived cardiomyocytes (iPSC-CMs) and H9C2 cells, leading to mitochondrial dysfunction and activation of necroptotic apoptotic pathways. Its overexpression significantly mitigates cellular injury (Chen et al., 2021; Yi et al., 2023). Mitophagy, while protective, may also exacerbate injury in radiation-induced heart disease (RIHD). It has been shown that nuclear dot protein 52 (NDP52)-induced mitophagy exacerbates inflammatory accumulation through the SH3-domain GRB2-like endophilin B1 (SH3GLB1)/PINK1 pathway, leading to myocardial injury. However, natural compounds such as Aloe vera rhododendron and Astragali polysaccharides exert protective effects by regulating the SH3GLB1/NDP52 axis, inhibiting autophagy activation, and modulating inflammatory responses (Gao et al., 2024; Ouyang et al., 2024; Jiang et al., 2025). This suggests that the effects of mitophagy are context-dependent, varying with tissue type and environmental conditions. Additionally, cuproptosis is strongly linked to mitochondria in RIHD. It has been observed that radiation upregulates copper transporter protein and chaperonin expression, facilitating copper ion entry into mitochondria, disrupting cytochrome c oxidase (cCO) assembly, impairing the function of the ETC, and inhibiting ATP synthesis. Notably, small extracellular vesicles (sEVs) derived from umbilical cord mesenchymal stem cells (UCMSCs) can reverse this process, alleviating copper-induced mitochondrial dysfunction and restoring energy metabolism (Cao H. et al., 2024).

Together, these findings highlight that cardiovascular injury caused by radiotherapy involves a complex interplay of mitochondrial dysfunction, disrupted energy metabolism, autophagy imbalance, and aberrant copper regulation, all of which are interconnected and influence each other. An in-depth understanding of these mechanisms will provide a solid theoretical foundation for the development of effective prevention and treatment strategies for RIHD.

### 5.4 Mitochondrial dysfunction in lung injury

Radiation-induced lung injury (RILI) is a common complication in patients undergoing chest radiotherapy for malignancies such as esophageal cancer, breast cancer, and thymoma (Yan et al., 2024), and the high radiosensitivity of lung tissue has become a key limiting factor in radiotherapy dose control. Excessive production of ROS induced by radiation triggers oxidative stress, inflammation, and apoptosis in alveolar epithelial cells, with mitochondrial dysfunction playing a central role in these pathological processes. Yang et al. reported that radiation-induced oxidative stress is accompanied by altered expression of mitochondria-related genes and mitochondrial depolarization, which subsequently activates the cGAS-STING signaling pathway (Yang et al., 2022), promoting the expression of pro-inflammatory cytokines such as NF-κB, TNF-α, and interleukin-6 (IL-6), and inducing immune cell infiltration (Chung et al., 2019). Mitochondrial oxidative stress also promotes macrophage polarization toward the pro-inflammatory M1 phenotype, leading to elevated cytokine secretion and amplifying the inflammatory microenvironment through a positive feedback loop (Yin et al., 2024). Moreover, epigenetic studies have shown that radiation induces methylation changes in genes regulating mitochondrial function, including Ras homolog family member T2 (RHOT2) and glutathione peroxidase 4 (GPX4). Although quantitatively limited, these modifications suggest a potential involvement of mitochondria in RILI progression via non-canonical pathways (Vera-Chang et al., 2023). Characterizing these genetic changes may offer novel therapeutic targets. A pronounced imbalance in mitochondrial quality control mechanisms is also evident in RILI. Radiotherapy disrupts the mitochondrial network in lung fibroblasts, and DRP1 dephosphorylation, along with interactions with mitochondrial fission proteins such as mitochondrial fission 1 protein (Fis1) and mitochondrial fission factor (Mff), promotes excessive mitochondrial fragmentation, which cooperates with stress protein p53 to induce cell death (Guo et al., 2022). Recently, reduced expression of the mitochondria-derived peptide MOTS-c has been associated with RILI severity. MOTS-c expression declines post-irradiation, whereas exogenous MOTS-c attenuates mitochondrial ROS production, preserves mitochondrial homeostasis, and mitigates alveolar epithelial cell apoptosis via activation of the Nrf2/ARE pathway, thereby alleviating radiation pneumonitis (Zhang et al., 2024b; 2024a).

Thus, the occurrence of RILI involves oxidative stress, inflammatory response, imbalance of mitochondrial dynamics and disruption of endogenous protective mechanisms. Targeted modulation of mitochondria-related pathways such as DRP1 inhibitors and MOTS-c analogs is expected to be a potential therapeutic strategy for future radiation lung injury.

### 5.5 Mitochondrial dysfunction in brain injury

Radiotherapy is one of the most effective treatments for primary and secondary brain tumors in adults and children (Liang et al., 2022). However, it is frequently accompanied by radiation-induced brain injury (RBI), which manifests as a range of neurological abnormalities, including learning and memory impairments, focal neurological deficits, increased intracranial pressure, secondary epilepsy, and progressive dementia (Turnquist et al., 2020). The central nervous system (CNS), composed of neurons and various types of glial cells, relies heavily on mitochondrial integrity and metabolic activity to maintain functional homeostasis (Geng et al., 2023). Radiotherapy not only directly causes DNA double-strand breaks in neurons but also activates the intrinsic mitochondrial apoptotic pathway, primarily regulated by p53. During this process, mitochondria release Cyt c and apoptosis-inducing factor (AIF-1). Cyt c translocates into the cytoplasm and activates downstream apoptotic signaling pathways, while the release of AIF-1 further accelerates the apoptotic program, ultimately resulting in neuronal apoptosis (Sabirzhanov et al., 2020).

Brain microvascular endothelial cells, as critical components of the blood-brain barrier (BBB), are particularly sensitive to mitochondrial oxidative stress, which significantly influences their survival. Targeted modulation of mitochondrial function in these cells can reduce apoptosis, preserve BBB integrity, alleviate neuroinflammation, and mitigate RBI-associated cognitive impairments (Zhang C. et al., 2023). In glial cells, mitochondrial dysfunction exhibits notable cell type specificity. Radiation-induced transformation of astrocytes into a neurotoxic A1 phenotype compromises neuronal repair and axonal regeneration; this process is closely associated with the collapse of MMP mediated by the outer membrane translocator protein (TSPO). TSPO modulators have been shown to stabilize membrane potential, enhance mitochondrial respiratory function, and reduce radiation-induced ROS production. These actions can reverse the pathological activation of A1 astrocytes, restore their proliferative capacity, and offer therapeutic potential for RBI (Zhang S. et al., 2023). Furthermore, microglia undergo mitochondrial metabolic reprogramming in response to radiation-induced stress, resulting in excessive mtROS production. These mtROS act as danger-associated molecular patterns (DAMPs), activating transcription factors such as specificity protein 1 (Sp1) and NF-κB, and inducing the expression of the sulfonylurea receptor 1–transient receptor potential melastatin 4 (SUR1-TRPM4) complex and NLRP3 inflammasomes, thereby initiating inflammatory responses. A chronic inflammatory environment hinders the differentiation of neural precursor cells into neurons, disrupts neural regeneration, and ultimately contributes to cognitive dysfunction (Chang et al., 2025).

The above findings reveal that radiotherapy disrupts CNS homeostasis through cell-specific mitochondrial dysfunction (MMP disturbance, ROS imbalance, metabolic reprogramming). Targeting key mitochondrial nodes, such as TSPO and mtROS, to regulate apoptosis and survival signaling and inhibit inflammatory responses may provide new strategies for RBI intervention.

### 5.6 Mitochondrial dysfunction in skin injury

Acute radiation dermatitis (ARD), a common complication of radiotherapy for breast cancer and head and neck tumors (Behroozian et al., 2023), involves a pathological process closely linked to mitochondrial dysfunction. Radiation impairs the mitochondrial function of skin cells through a threefold mechanism: ① directly inducing the collapse of MMP, triggering calcium overload and the abnormal activation of transient receptor potential melastatin 2 (TRPM2) channels, leading to the loss of fibroblast proliferation and migration (Huangfu et al., 2022); ② disrupting redox homeostasis, as evidenced by the elevation of ROS/malondialdehyde (MDA) levels and the inhibition of the antioxidant enzyme system, including superoxide dismutase (SOD) and catalase (CAT) (Huangfu et al., 2022), and ③ interfering with mitochondrial energy metabolism by decreasing the efficiency of ATP synthesis and hindering the process of re-epithelialization (Du et al., 2022). These alterations create a vicious cycle—mitochondrial damage exacerbates oxidative stress, which, in turn, further amplifies mitochondrial dysfunction, ultimately leading to epidermal barrier disruption and delayed dermal repair. Consistent with this, Whitcomb et al. found that protracted γ-ray exposure increased fibroblast mitochondrial oxygen consumption and H_2_O_2_ release rates, along with activation of gene programs related to oxidative stress, which were attenuated by the mitochondria-specific antioxidant mitoTEMPO (Whitcomb et al., 2024). In terms of intervention strategies, radioprotective agents targeting mitochondria show remarkable potential. For instance, α2-macroglobulin (α2-M) repairs radiation damage through a dual mechanism—restoring mitochondrial calcium homeostasis and reestablishing antioxidant enzyme defenses (Huangfu et al., 2022). Additionally, the mitochondria-specific drug CY-TMP1 promotes vascularization and epithelial regeneration by scavenging mtROS, maintaining MMP and ATP production, and promoting neovascularization and epithelial regeneration (Du et al., 2022).

Furthermore, stem cell-based therapeutic strategies have demonstrated potential. Studies show that transferring mitochondria-rich extracellular vesicles into skin-damaged tissues improves mitochondrial dysfunction, reconfigures energy metabolism networks in damaged cells, and ameliorates the inflammatory microenvironment by modulating the macrophage M1/M2 polarization balance (Yao et al., 2024). Together, these findings reveal the central regulatory position of mitochondria in radiodermatitis and provide a theoretical basis for the development of precise protection programs.

Collectively, current evidence suggests that mitochondrial dysfunction represents a unifying yet organ-specific feature of radiation-induced tissue injury. In radiation-sensitive organs—including the intestine, hematopoietic system, cardiovascular system, lungs, brain, and skin—disruptions in mitochondrial dynamics, bioenergetics, redox homeostasis, and cell survival pathways contribute significantly to tissue damage. Although common mechanisms such as oxidative stress, mtDNA damage, and impaired energy metabolism are broadly observed, each organ exhibits unique mitochondrial vulnerabilities and therapeutic targets, as summarized in Table 1. This comprehensive overview not only highlights the central role of mitochondrial dysfunction in radiation pathology but also provides a mechanistic basis for the therapeutic strategies discussed in the subsequent section.

**TABLE 1:** Summary of organ-specific mitochondrial dysfunction manifestations, mechanisms, and potential therapeutic strategies following radiation exposure.

Organ	Mitochondrial dysfunction manifestation	Key mechanisms	Potential therapeutic strategies	References
Intestinal	ATP ↓^a^ ROS ↑^b^ MtDNA damage	Caspase-3/9 activation ETC activity inhibition Suppressed mitophagy (PINK1, Parkin downregulation)	Mitophagy activators (rapamycin) Immune signaling activators (MOS) Antioxidants (Hydrogen)	Qu et al. (2020) Sanguri and Gupta (2021) Qiu et al. (2020)
Hematopoietic System	ROS ↑ Disruption of MMP MtDNA damage	Increased frequency and duration of mPTP opening; activated cGAS-STING pathway; Reduced serum serine levels	Inhibition of mtDNA release or cGAS activation Natural antioxidant and specific 5-LOX inhibitor (caffeic acid) Exogenous serine supplementation Nynrin overexpression	Guan et al. (2023) Wang X. et al. (2021) Du et al. (2024) Zhou et al. (2024)
Cardiovascular	ROS ↑ Disruption of MMP Impaired OXPHOS MtDNA damage Disrupted cCO assembly	Mitochondrial calcium overload TGF-β activation promoting fibrosis Cuproptosis	Statins (pravastatin) Stem cell therapy (UCMSC exosomes) PTPMT1 overexpression	Ait-Aissa et al. (2023) Cao H. et al. (2024) Chen et al. (2021), Yi et al. (2023)
Lung	ROS ↑ Mitochondrial dynamic imbalance (DRP1 dephosphorylation)	activated cGAS-STING pathway Altered mitochondrial gene methylation	Nrf2/ARE pathway modulators (MOTS-c)	Zhang et al. (2024b), 2024a
Brain	ROS ↑ Disruption of MMP Mitochondrial metabolic reprogramm	p53-mediated mitochondrial apoptosis pathway NLRP3 inflammasome activation A1 astrocyte transformation Impaired neural regeneration	TSPO modulators NLRP3 inhibitors	Zhang S. et al. (2023) Chang et al. (2025)
Skin	ATP ↓ ROS ↑ Disruption of MMP	TRPM2 channel abnormal activation Redox imbalance ATP synthesis impairment	α2-macroglobulin (α2-M) The mitochondria-specific drug (CY-TMP1) Stem cell therapy (exosome delivery)	Huangfu et al. (2022) Du et al. (2022) Yao et al. (2024)

^a^ ATP ↓:Decreased ATP, production.

^b^ ROS ↑:Excessive ROS, generation.

## 6 Targeted mitochondrial therapeutic strategies in radiation tissue injury

**FIGURE 4:**
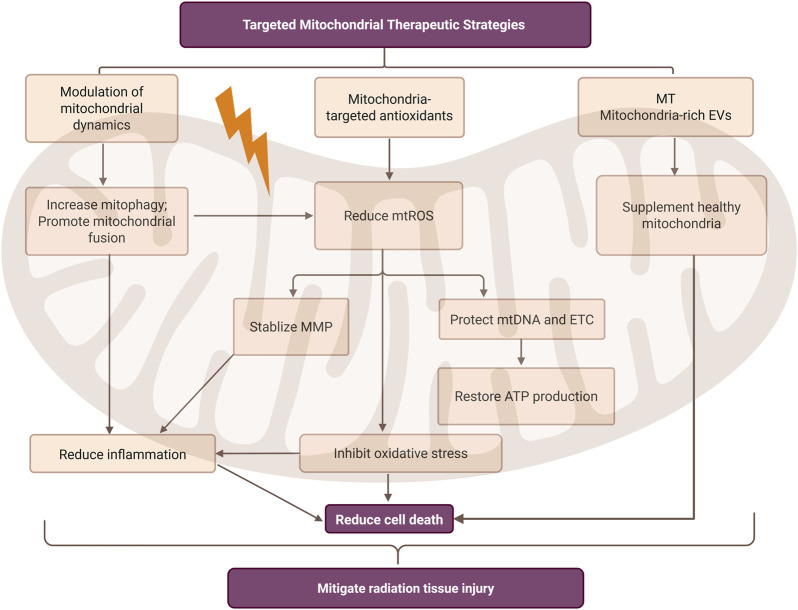
Mitochondria-targeted therapeutic strategies in radiation-induced tissue injury. This figure summarizes three main mitochondrial intervention measures: mitochondrial dynamic regulation, mitochondrial-targeted antioxidants, and emerging regenerative therapies. These strategies aim to reduce mitochondrial reactive oxygen species (mtROS), stabilize mitochondrial membrane potential (MMP), and restore ATP production, thereby ultimately alleviating inflammatory responses, inhibiting oxidative stress, and reducing cell death. It can also be observed that there is a mutual correlation between reducing inflammatory responses and inhibiting oxidative stress. MT, mitochondrial transplantation; EVs, extracellular vesicles. Created in BioRender. rong, j. (2025) https://BioRender.com/1hc2h6e.

Radiation-induced tissue injury remains a major challenge in cancer radiotherapy, with few effective therapies targeting mitochondrial dysfunction. Advances in understanding radiation-induced mitochondrial pathology have revealed several promising strategies, including modulation of mitochondrial dynamics, application of mitochondria-targeted antioxidants, and emerging regenerative approaches such as mitochondrial transplantation and mitochondria-rich extracellular vesicles (Figure 4).

### 6.1 Modulation of mitochondrial dynamics

Mitophagy, a key component of mitochondrial dynamics, has emerged as a promising therapeutic target for radiation-induced tissue injury (Wu et al., 2017). Strict mitochondrial quality control systems are essential to maintain mitochondrial integrity and function (De et al., 2021; Kumar Sharma et al., 2022). Experimental studies have demonstrated that inhibiting mitophagy—via pharmacological inhibitors or genetic knockdown of Parkin—leads to elevated ROS production and aggravated nuclear DNA damage, ultimately increasing radiation-induced cell death (Yang et al., 2021). In a murine model of radiation-induced kidney injury, enhanced mitophagy attenuated NLRP3 inflammasome activation and apoptosis, highlighting its protective role (Ding et al., 2024). Beyond targeting the classical PINK1/Parkin axis, other regulators have been identified. Spata18, a transcriptional target of p53, facilitates mitophagy following DNA damage. Spata18 knockdown significantly reduced mitophagy and compromised DNA repair capacity in fibroblasts (Dan et al., 2020). In parallel, mitochondrial fusion and fission dynamics also critically influence the cellular response to radiation stress. G protein-coupled receptor kinase 2 (GRK2), a stress-responsive kinase, promotes mitochondrial fusion by phosphorylating MFN1 and MFN2, thereby alleviating oxidative stress and maintaining mitochondrial integrity (Franco et al., 2018).

These research results indicate that mitophagy is a feasible therapeutic target in radiation-induced damage. By regulating key regulatory factors such as PINK1, Parkin, and Spata18, oxidative stress can be alleviated, inflammation can be inhibited, and cell death can be reduced. Moreover, mitochondrial fusion mediated by GRK2 may help maintain the integrity and redox balance of mitochondria. These complementary strategies collectively provide a promising foundation for mitochondrial-targeted interventions against tissue damage caused by radiation.

### 6.2 Mitochondria-targeted antioxidants

As discussed in earlier sections, mitochondria are major sources of intracellular ROS, and their dysfunction plays a central role in radiation-induced oxidative stress (Wang M. et al., 2023). mtDNA, lacking histone protection and possessing limited DNA repair capacity, is particularly susceptible to oxidative damage, leading to disruption of the OXPHOS pathway and impaired ATP production (Wang Q-Q. et al., 2021). Furthermore, excessive radiation-induced ROS can damage mitochondrial membranes, trigger the opening of the mitochondrial permeability transition pore (mPTP), and decrease MMP, collectively compromising ETC function and energy metabolism (Duan et al., 2022). As a result, antioxidant strategies specifically targeting mitochondria have garnered increasing attention. In addition to the mitochondria-targeted antioxidants summarized in Table 1, Mitoquinone (MitoQ)—a derivative of coenzyme Q10 (CoQ10)—is a commercially available mitochondrial-targeted antioxidant designed to selectively mitigate oxidative stress within mitochondria. Although MitoQ has not yet been evaluated in the context of radiation-induced injury, several clinical trials have investigated its potential in other human conditions. For instance, a completed clinical trial (ClinicalTrials.gov ID: NCT03506633) assessed the effects of MitoQ on oxygen delivery capacity, skeletal muscle mitochondrial function, leg performance, and claudication in patients with peripheral artery disease. Another ongoing trial (ClinicalTrials.gov ID: NCT04851288) is examining its ability to improve vascular endothelial function in older adults. Although these trials are not radiation-specific, they underscore the translational relevance of mitochondria-targeted antioxidants in protecting mitochondrial structure and redox function, thereby supporting their potential application in radiation-exposed tissues.

### 6.3 Emerging regenerative approaches for mitochondrial restoration

Radiation-induced mitochondrial dysfunction in normal tissues may potentially be reversed through mitochondrial transplantation. This emerging regenerative strategy has shown great promise in treating various diseases associated with mitochondrial impairment. By introducing healthy mitochondria into damaged cells, this approach enhances ATP production, reduces ROS accumulation, modulates inflammation, and suppresses apoptosis, thereby promoting tissue repair and regeneration (Li et al., 2025). Recent studies have demonstrated that mitochondria-rich extracellular vesicles derived from stem cells can serve as carriers to deliver healthy mitochondria into radiation-injured skin tissues, effectively promoting wound healing and tissue regeneration (Yao et al., 2024). Although direct evidence in radiation injury models remains limited, the demonstrated efficacy of mitochondrial transplantation in other pathological contexts suggests its potential as a novel therapeutic avenue for restoring mitochondrial function and promoting tissue repair following radiation-induced injury.

## 7 Summary and outlook

Mitochondria are not only the center of cellular energy metabolism but also a major target of radiation-induced tissue damage. Radiation induces mtDNA damage, impairs mitochondrial dynamics, defects in the ETC, and alters energy metabolism, leading to mitochondrial dysfunction, which in turn triggers oxidative stress, apoptosis, and affects tissue function (Abate et al., 2020). An increasing amount of evidence currently indicates that maintaining the functional homeostasis of mitochondria after radiation plays a crucial role in alleviating radiation-induced tissue injury.

However, these studies are still at the cellular or animal experiment stage and lack verification of clinical relevance. Moreover, existing studies mainly focus on a single intervention pathway (such as anti-oxidation or autophagy activation), and there is still a relative shortage of research on multi-pathway collaborative intervention strategies. In addition, the targeted intervention strategies for the radiation-induced damage of mitochondria are still in the initial exploration stage, and there is a lack of effective and translatable intervention methods and drugs. Except for mature red blood cells, mitochondria are present in the vast majority of cells. In the future, in order to further enhance the understanding of the connection between mitochondrial dysfunction and radiation-induced tissue damage, the following directions should be focused on: Firstly, strengthen the research on the cross-regulatory mechanisms between mitochondrial signaling pathways and other cellular stress networks (such as immune responses, endoplasmic reticulum stress, etc.). Secondly, develop more precise and effective mitochondrial-targeted protection strategies, considering multi-pathway synergistic targeting effects. For example, combine mitochondrial-targeted antioxidants, membrane potential regulators, or autophagy regulatory molecules, etc. Thirdly, promote the clinical transformation of basic research achievements, establish mitochondrial-targeted intervention strategies for radiation protection and tissue repair, achieve a closed loop from mechanism exploration to intervention application, reconstruct the physiological functions of mitochondria under radiation, and restore the equilibrium state of cells.
