# Correction: Advances in mitochondrial dysfunction in radiation tissue injury

**DOI:** 10.3389/fphys.2025.1690425

**Published:** 2025-09-02

**Authors:** Jianhuang Rong, Qiujie Yu, Guilin Huang, Yueyue Wang, Nini Zhang

**Affiliations:** ^1^ Department of Oral Maxillofacial Surgery, School and Hospital of Stomatology, Zunyi Medical University, Zunyi, China; ^2^ Department of Laboratory Medicine, The Third Affiliated Hospital of Zunyi Medical University (The First People’s Hospital of Zunyi), Zunyi, China; ^3^ Zunyi Medical University, Zunyi, China

**Keywords:** mitochondrial dysfunction, radiation injury, oxidative stress, mitophagy, apoptosis, energy metabolism

In the published article the affiliation of the authors Nini Zhang and Qiujie Yu were wrongly assigned.

“Author Nini Zhang was erroneously assigned to affiliation “Department of Laboratory Medicine, The Third Affiliated Hospital of Zunyi Medical University (The First People’s Hospital of Zunyi), Zunyi, China”. The correct affiliation of Author Nini Zhang should be “Department of Oral Maxillofacial Surgery, School and Hospital of Stomatology, Zunyi Medical University, Zunyi, China”.”

“Author Qiujie Yu was erroneously assigned to affiliation “Zunyi Medical University, Zunyi, China”. The correct affiliation of Author Qiujie Yu should be “Department of Laboratory Medicine, The Third Affiliated Hospital of Zunyi Medical University (The First People’s Hospital of Zunyi), Zunyi, China”.”

The original article has been updated.

